# CARD14/CARMA2 Signaling and its Role in Inflammatory Skin Disorders

**DOI:** 10.3389/fimmu.2018.02167

**Published:** 2018-09-26

**Authors:** Tiziana Zotti, Immacolata Polvere, Serena Voccola, Pasquale Vito, Romania Stilo

**Affiliations:** ^1^Genus Biotechnology, Università degli Studi del Sannio, Benevento, Italy; ^2^Dipartimento di Scienze e Tecnologie, Università degli Studi del Sannio, Benevento, Italy

**Keywords:** CARD14, CARMA2, NF-kappa B, psoriasis, Bcl10, Malt1, CBM complex

## Abstract

CARMA proteins represent a family of scaffold molecules which play several crucial biological functions, including regulation of immune response and inflammation, tissue homeostasis, and modulation of G-Protein Coupled Receptor (GPCR) signaling. Among the CARMA proteins, CARD14/CARMA2 and its alternatively spliced isoforms are specifically expressed in epithelial cells and keratinocytes. Recent evidences have shown that CARD14/CARMA2 mediates induction of inflammatory response in keratinocytes, and that mutations in CARD14/CARMA2 gene segregate with familial transmission of chronic inflammatory disorders of the human skin. Similarly to CARD11/CARMA1 and CARD10/CARMA3, CARD14/CARMA2 signaling occurs trough formation of a trimeric complex which includes BCL10 and MALT1 proteins. However, it is becoming increasingly evident that in addition to the CBM complex components, a number of accessory molecules are able to finely modulate the signals conveyed on and amplified by CARD14/CARMA2. The study of these molecules is important both to understand the molecular mechanisms that underlie the role of CARMA2 in keratinocytes and because they represent potential therapeutic targets for the development of therapeutic strategies aiming at the treatment of inflammatory diseases of the human skin. In this review, we provide an overview on the molecular mechanisms mediating CARD14/CARMA2 signaling and its implication in our understanding of the pathogenesis of human inflammatory skin disorders.

## CARMA family: an overview

**Figure 1:**
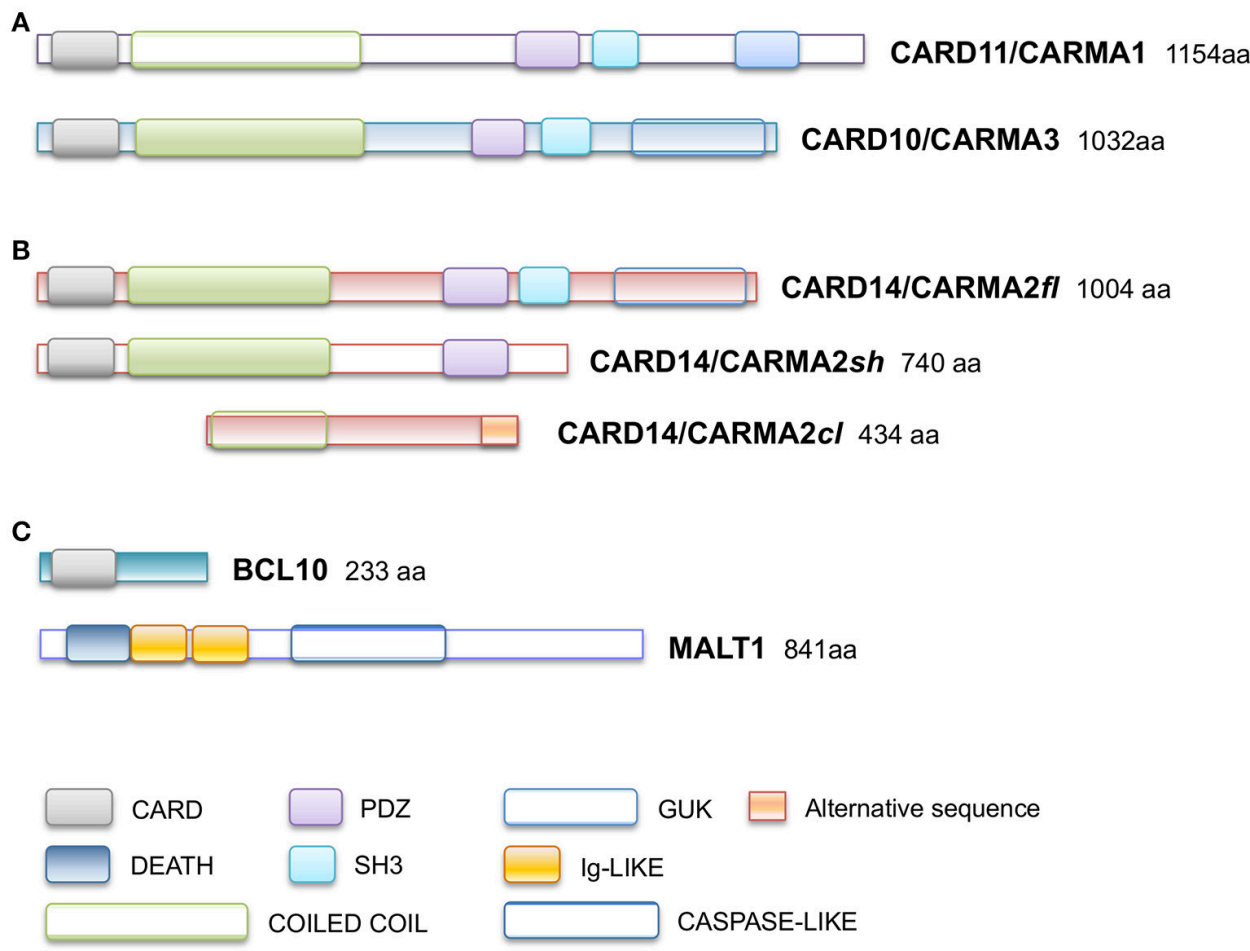
Schematic representation of domain organization of **(A)** CARD11/CARMA1 (UniProt Entry: UniProt) and CARD10/CARMA3 (UniProt Entry: Q9BWT7) proteins, **(B)** CARD14/CARMA2 isoforms (UniProt Entry: Q9BXL6) **(C)** BCL10 (UniProt Entry: O95999) and MALT1 proteins (UniProt Entry: Q9UDY8). Aminoacid numbering and domains are referred to human proteins. CARD, CAspase-Recruitment Domain; PDZ, post-synaptic density protein (PSD95), Drosophila disc large tumor suppressor (Dlg1), and Zonula occludens-1 protein (Zo-1) domain; GuK, guanylate kinase-like; DEATH, death domain; SH3, Src homology 3; Ig-LIKE, immunoglobulin-like domain; COILED COIL, coiled coil region; CASPASE-LIKE, Cysteine-ASpartic ProteASEs-like domain.

Caspase recruitment domain (CARD)-containing membrane-associated guanylate kinase (MAGUK) proteins constitute a family of three scaffold proteins, highly conserved in their amino acidic sequence, named CARD11/CARMA1 (CARMA1), CARD14/CARMA2 (CARMA2), and CARD10/CARMA3 (CARMA3) (1, 2). CARMA proteins were identified in 2001, while screening sequence databases and two-hybrid libraries for novel CARD-containing proteins, and were shown to be able to interact with the CARD domain of B-Cell Leukemia 10 (BCL10) (3–6). The human CARMA proteins are encoded by three conserved genes, respectively located on chromosomes 7, 17, and 22. Structurally, CARMA proteins are characterized by a typical modular organization, with the CARD domain at the N-terminus, followed by a Coiled-Coil region and a C-terminal MAGUK domain, consisting of PDZ, SH3, and GUK modules (Figure 1A). Despite the high degree of structural similarity, the expression pattern of each CARMA protein is restricted to distinct tissues, where they are involved in cell-specific signaling pathways that control activation of NF-κB, a pleiotropic transcription factor that controls transcription of, among others, immunomodulatory and inflammatory genes and genes that generally promote cell proliferation and survival (7). Indeed, CARMA1 is mainly expressed in lymphoid cells and hematopoietic tissues, where it mediates NF-κB induction following antigen receptor engagement. Notably, CARMA1 deficient-mice show a severe defect in lymphocyte proliferation following T- and B-cell receptor stimulation, with impaired production and release of cytokines (8–10), due to defective NF-κB activation. Consistently with evidences from animal models, whereas loss-of-function mutations in human *CARMA1* gene cause severe forms of immunodeficiencies (11–13), gain-of-function mutations have been frequently described in patients affected by diverse lymphoid malignancies, such as T-cell lymphomas, gastric B-cell lymphomas, some non-Hodgkin's lymphomas and others (14).

Conversely, CARMA2 and CARMA3 are both expressed in non-lymphoid tissues, but in a non-overlapping manner. In fact, CARMA2 protein is preferentially expressed in epithelial cells of the skin and in mucosae, while CARMA3 has a broader non-hematopoietic expression pattern (2). In these districts, CARMA3 regulates NF-κB activation following stimulation of G-protein coupled receptors (GPCRs) with several ligands, such as angiotensin II, endothelin I, and lyso-phosphatidic acid (15, 16). In addition, CARMA3 has also been shown to be involved in NF-κB activation downstream of the epithelial growth factor receptor (17). Not surprisingly, CARMA3 over-expression has been shown to be implicated in the onset and progression of different cancers by several studies (18, 19).

Compared to CARMA1 and CARMA3, CARMA2 is less characterized. Only recent studies have shed light on the crucial role this scaffold protein plays in the human skin, where it regulates tissue homeostasis.

## CARMA2: cloning, splice variants, expression

CARMA2 was originally identified as a placenta-specific cytoplasmic 1,004 amino acids protein containing a CARD module and a MAGUK domain, and capable to activate the NF-κB-controlled expression of a luciferase reporter gene when transfected in cultured cell lines (3). Subsequent works have demonstrated that *CARMA2* mRNA undergoes alternate splicing processes (20) and that the deriving protein isoforms show a wider distribution profile, being expressed also in epidermal keratinocytes, dermal endothelial cells, mucosae and different cell lines (20, 21). In particular, three transcript variants of human *CARMA2* gene have been identified, named CARMA2*fl* (*full length*; 1,004 amino acids), the longest polypeptide containing all the typical CARMA domains and modules; CARMA2*sh* (*short*; 740 amino acids), the prominent isoform expressed in the human skin, lacking the SH3 and GuK modules and containing the CARD, coiled coil and PDZ domains; and CARMA2*cl* (*cardless*; 434 amino acids), containing only a portion of the coiled coil domain, the linker region and a shorter PDZ module (Figure 1B). Due to the absence of a complete MAGUK domain, CARMA2*cl* and CARMA2*sh* variants may not be exclusively associated to the cell membrane, but rather distributed in the cytosol where they possibly transduce intracellular signals (20).

As assessed by NF-κB-luciferase reporter assays, while CARMA2*fl* and especially CARMA2*sh* are strong inducers of NF-κB, CARMA2*cl* is unable to promote activity of this transcription factor (20), confirming the CARD region as an essential domain for NF-κB signaling regulated by CARD-containing proteins. Indeed, similarly to CARMA1 and CARMA3, CARMA2*fl*, and CARMA2*sh*, but not CARMA2*cl*, interact with BCL10 via an homotypic CARD-CARD association and, together with the paracaspase Mucosa Associated lymphoid tissue Lymphoma Translocation protein 1 (MALT1), are able to form a CARMA-BCL10-MALT1 (CBM) complex (Figure 1C). Assembly of the CBM complex is crucial for the recruitment of downstream signaling components that lead to NF-κB activation (20, 22, 23). Differently from the longest CARMA2 isoforms, CARMA2*cl* has a very limited expression profile in non-epidermal tissue, and in transfection experiments it may function as a natural dominant-negative regulator of CARMA2*sh* signaling in the skin (Scudiero and Vito, unpublished results). Overall, the identification of alternative transcripts for *CARMA2* is intriguing for several reasons: first, their expression pattern is not totally overlapping, suggesting that they could play diverse functions in different cell types; second, even within the same cell type, *CARMA2* transcript variants could regulate different stimuli, starting both from the cell membrane and intracellular organules, or regulate signals from the same stimulus at several levels.

## CARMA2 in keratinocytes and skin disorders

Psoriasis is an inflammatory disorder of the human skin, characterized by well-demarcated oval-shaped erythematous plaques on the skin due to abnormal keratinization and proliferation of superficial keratinocytes, and persistence of nucleated cells in the corneus layer (24). The onset of psoriasis depends on both genetic and environmental factors and is characterized by the disruption of the epithelial barrier function and tissue homeostasis due to stressing or traumatic events within the epidermis, and a dysregulated immune response. Epidemiologic studies based on data collected on psoriatic patients from 20 different countries show that psoriasis is a complex genetic-based immune-mediated disease with a prevalence ranging from 0 to 1.37% in children and from 0.51 to 11.43% in adults, with at least 100 million individuals affected worldwide (25). Indeed, in the human genome multiple susceptibility loci, collectively called *PSORS*s, have been associated to the familial transmission of the psoriatic tract, with the identification of about 40 genes involved in antigen presentation, interleukin and cytokine signaling, antiviral response, NF-κB signaling and, more generally, in the adaptive and innate immunity (24). Nevertheless, although a genetic base underlies psoriasis and psoriasis-related diseases, <20% of disease variance is explained by mutations in the aforementioned genes, suggesting the existence of additional mechanisms which could trigger these skin inflammatory pathologies (26).

In 2012, Jordan et al. established that *PSORS2* was due to gain-of-function mutations in the *CARD14* gene, as assessed by exome capture and next generation sequencing over genomic DNA from both familial and sporadic cases of psoriasis and psoriatic arthritis (27, 28). Most of the psoriasis-linked mutations harbored in CARMA2*sh* produced an enhanced activity of NF-κB transcription factor in luciferase assays, with a consequent up-regulation of NF-κB-induced inflammatory transcripts in keratinocytes, such as CXCL8, CCL20, IL8, and IL6, confirming the crucial role played by this transcription factor in epithelial homeostasis (27, 28). In addition to genetic psoriasis, mutations in *CARMA2* were also found in familial cases of pityriasis rubra pilaris, a papulosquamous disorder phenotypically related to psoriasis (29).

**Figure 2:**
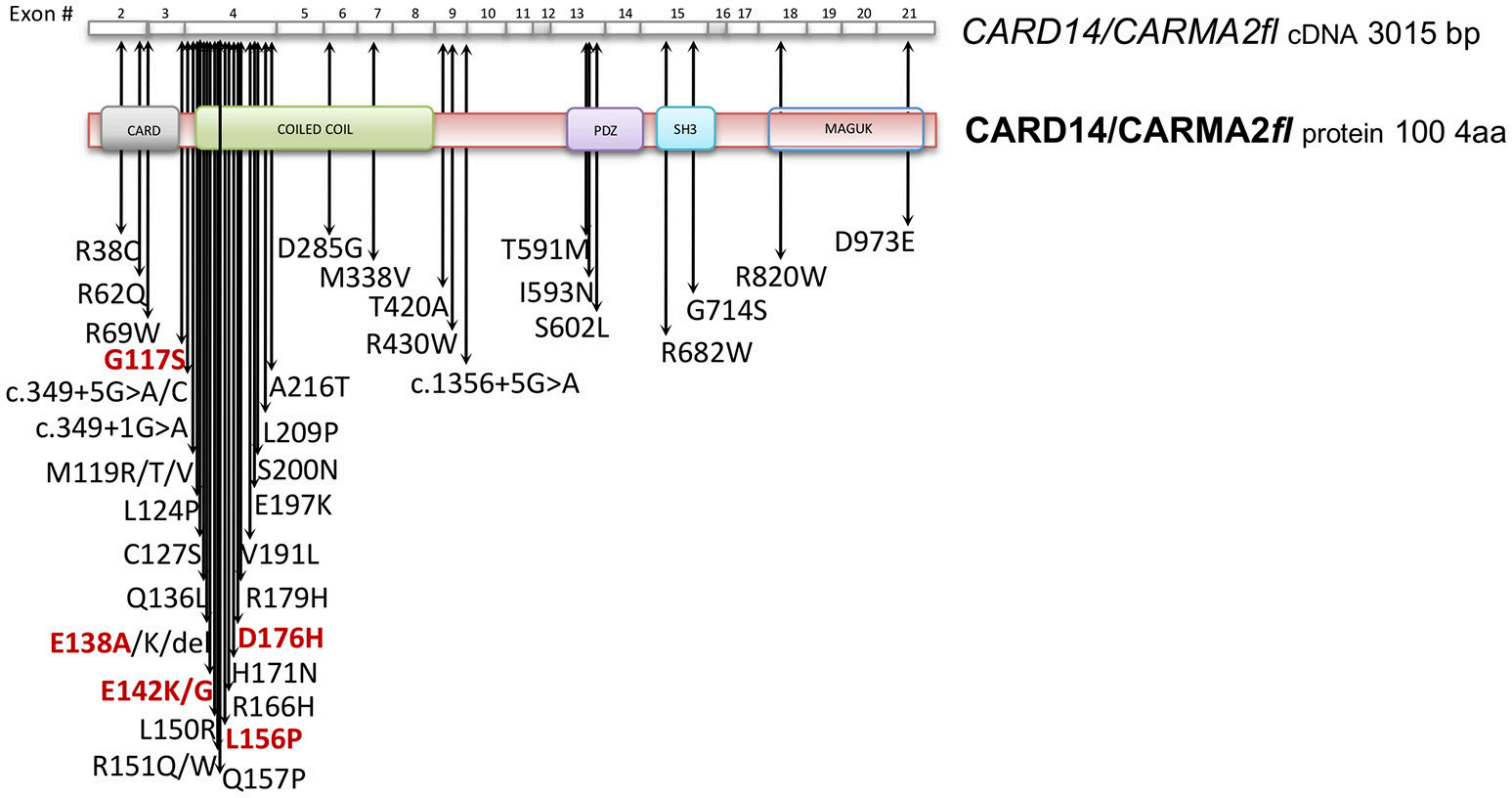
CARD14/CARMA2 variants associated to skin inflammatory diseases (see also Table 1). Arrows indicate mutated base positions within CARD14/CARMA2 exons and corresponding aminoacid substitution identified in psoriasiform patients. In red, missense mutations having a reported positive effect on NF-κB activation.

Subsequently, plenty of sequence variations and mutations in the *CARD14* gene have been mapped and associated to psoriasis, pityriasis and other skin disorders phenotipically related to them. Table 1 reports all known CARMA2*sh* variants associated to skin inflammatory illnesses that have been identified so far, updating a list already provided by Van Nuffel et al. (50). Figure 2 show that base mutations preferentially involve codons in exon 4, resulting in amino acidic substitutions within the CARD and Coiled-Coil domains.

**Table 1:** List of CARMA2 variants associated to psoriasis or psoriasiform inflammatory diesases.

**Mutation**	**Disease**	**References**
R38C	Psoriasis vulgaris	(27)
R62Q	Psoriasis vulgaris	(27, 30)
R69W	Psoriasis vulgaris	(30)
G117S	Psoriasis vulgaris; Pityriasis rubra pilaris	(27, 28, 30–33)
c.349 + 5G > A	Psoriasis vulgaris	(27, 28)
c.349 + 5G > C	Psoriasis; Pityriasis rubra pilaris	(34)
c.349 + 1G > A	Pityriasis rubra pilaris; Generalized pustular psoriasis	(29, 35)
M119R	Pityriasis rubra pilaris	(36)
M119T	Psoriasis; Pityriasis rubra pilaris	(34)
M119V	Generalized pustular psoriasis	(37)
L124P	Pityriasis rubra pilaris	(38)
C127S	Pityriasis rubra pilaris	(33)
Q136L	Pityriasis rubra pilaris	(33)
E138A	Generalized pustular psoriasis	(27, 28)
E138K	Pityriasis rubra pilaris	(39, 40)
E138del	Pityriasis rubra pilaris	(29)
E142K	Psoriasis vulgaris	(27, 28)
E142G	Psoriasis vulgaris	(27, 28)
L150R	Psoriasis vulgaris	(27, 30)
R151W	Psoriasis vulgaris	(30)
R151Q	Psoriasis vulgaris	(30)
L156P	Pityriasis rubra pilaris	(29)
Q157P	Psoriasis; Pityriasis rubra pilaris	(34)
R166H	Generalized pustular psoriasis	(37)
H171N	Psoriasis vulgaris	(27)
D176H	Psoriasis vulgaris; Generalized pustular psoriasis; Pityriasis rubra pilaris	(27, 41–45)
R179H	Psoriasis vulgaris; Pityriasis rubra pilaris	(27, 43)
V191L	Psoriasis vulgaris	(27)
E197K	Psoriasis vulgaris; Pityriasis rubra pilaris; Psoriatic arthritis	(30, 43)
S200N	Psoriasis vulgaris; Generalized pustular psoriasis; Pityriasis rubra pilaris	(27, 30, 31, 43)
L209P	Psoriasis vulgaris	(30)
A216T	Psoriasis vulgaris	(30, 37, 44)
D285G	Psoriasis vulgaris	(27)
M338V	Psoriasis vulgaris	(30)
T420A	Psoriasis vulgaris	(30)
R430W	Acute generalized exanthematous pustulosis	(46)
c.1356 + 5G > A	Psoriasis vulgaris	(30)
T591M	Psoriasis vulgaris	(37)
I593N	Psoriasis vulgaris	(27)
S602L	Psoriasis vulgaris; Generalized pustular psoriasis; Pityriasis rubra pilaris	(30)
R682W	Psoriasis vulgaris; Generalized pustular psoriasis	(27, 37)
G714S	Psoriasis vulgaris	(27)
R820W	Psoriasis vulgaris; Psoriatic arthritis	(27, 47–49)
D973E	Psoriasis vulgaris	(27)

Although many of the *CARMA2* mutations found in a variety of inflammatory disorders of the human skin point to an aberrant activation of NF-κB, it is worth noting that some patients carry mutations in CARMA2*sh* (such as Arg38Cys; Arg69Trp; Arg151Trp; His171Asn; Ser200Asn; Ala216Thr; Thr420Ala) that, at least in luciferase-based *in vitro* assays, do not determine a remarkably stronger activation of NF-κB (28, 30, 50), suggesting that CARMA2*sh* could participate to additional intracellular mechanisms regulating skin homeostasis. In fact, other studies have demonstrated that several CARMA2*sh* variant, including Arg820Trp, could significatively affect the response to anti-TNFα treatment in psoriasis patients, with interesting implications for optimal therapy settings (51, 52).

## CARMA2 signaling

**Figure 3:**
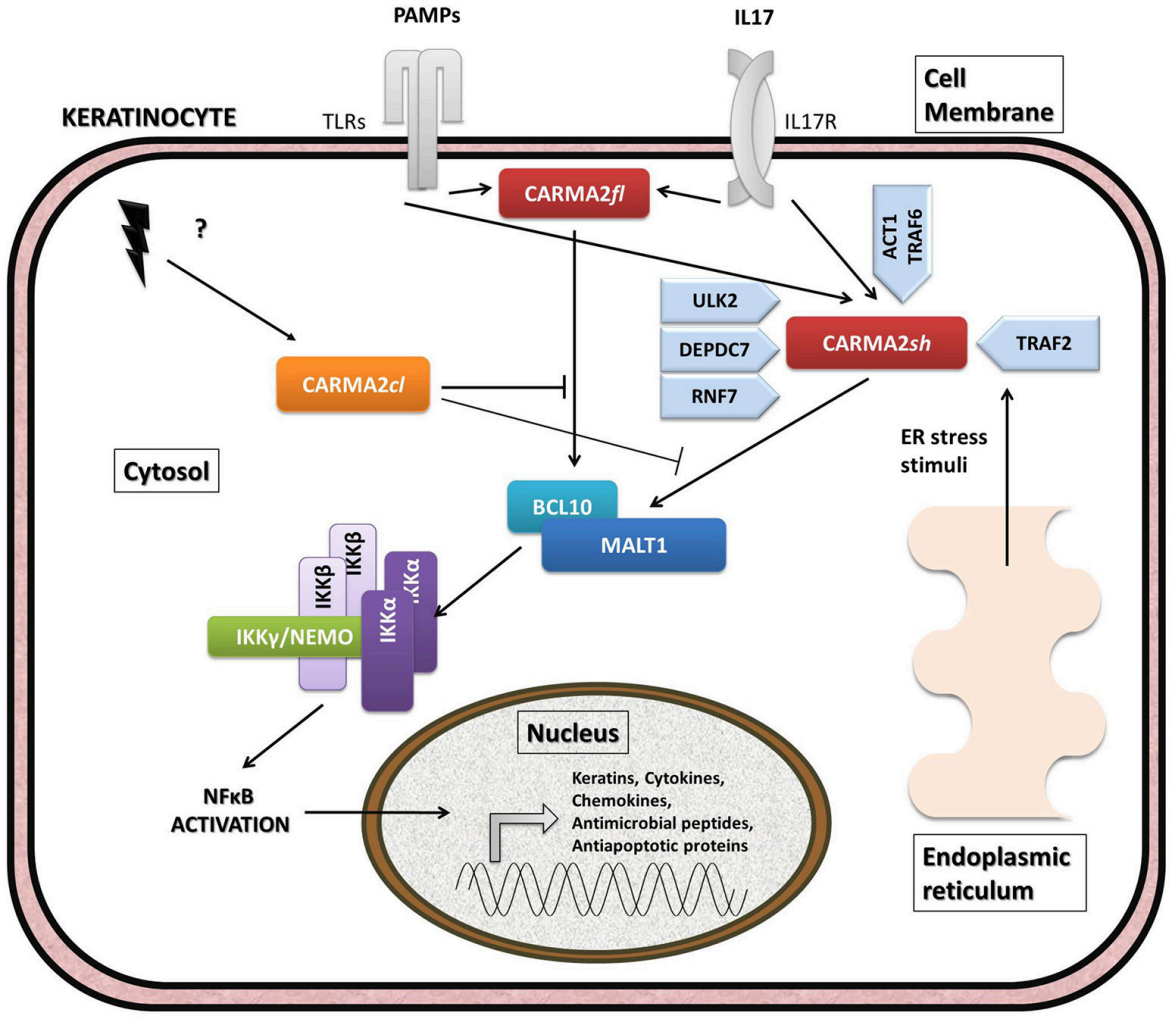
Schematic overview of intracellular pathways involving CARD14/CARMA2 variants in keratinocytes. In the skin, CARMA2-mediated signals are prompted by intracellular organelles, such as endoplasmic reticulum, and by surface receptors, such as Toll-Like Receptors (TLRs) and IL17-Receptor (IL17R) in response to Pathogen Associated Membrane Patterns (PAMPs) and IL17, respectively. While CARMA2cl would act as a natural negative regulator, CARMA2sh and CARMA2fl variants interact with BCL10 and MALT1, thereby forming CBM complex. In turn, CBM triggers a canonical NEMO-dependent NF-κB-activation pathway, mediated by the IκB Kinase complex (IKK complex), which leads to expression of target genes in the nucleus, such as keratins, antimicrobial compounds and inflammatory mediators. Negative and positive regulators of CARMA2sh identified so far are also reported in the figure.

Given its involvement in the pathogenesis of psoriasis, many efforts have focused on the understanding of the molecular mechanisms through which CARMA2*sh* regulates signaling cascades in human keratinocytes (Figure 3). Experimental data indicate that CARMA2*sh* signaling requires assembly of a molecular complex that, in addition to CARMA2*sh*, also includes the adapter protein BCL10 and the protease MALT1, as demonstrated by experiments conducted using short interfering RNAs, genome editing methods and chemical inhibitors (20, 22, 53). Most of the details that control assembly of the CBM complex and its activation derive from data obtained with CARMA1 in lymphocytes. In un-stimulated cells, CARMA1 is held in check by the inhibitory linker region, located between the coiled-coil domain and the PDZ domain. Following cell stimulation, such auto-inhibition is removed through PKCs-mediated phosphorylation of serine residues in the linker region, thereby facilitating BCL10 and MALT1 binding to the CARMA1 (54, 55). The assembly of the CBM complex eventually results in the recruitment of the IKK complex and the consequent NF-κB activation (23). The similarity of the mechanisms of activation of the different CARMA proteins is also suggested by the fact that ectopic expression of the deubiquitinase A20 inhibits activation of NF-κB mediated by each of the CARMA proteins (20, 22, 56). Consistently with the model that considers assembly of the CBM complex as a crucial point for activation of the NF-κB pathway, two highly penetrant psoriasis-linked CARMA2*sh* point mutations, Gly117Ser, and Glu138Ala, abrogate CARMA2 auto-inhibition and stimulate MALT1 protease activity, causing constitutive activation of the CBM complex and aberrant NF-κB-dependent induction of downstream inflammatory genes (22, 53). Interestingly, MALT1 deficiency or chemical inhibition of its catalytic activity can block hyperactivation of the inflammatory signaling program (including the induction of TNFα, IL17C, CXCL8, and HBD2 genes), triggered by pathogenic psoriasis-related CARMA2*sh* mutants or by cell stimulation with the fungal cell wall component zymosan or with *Staphilococcus aureus*, pointing to MALT1 as a potential target for therapeutic treatment of skin disorders caused by aberrant CARMA2*sh* signaling (22, 50, 53, 57). In addition to the component of the CBM complex, the NF-κB-inducing activity of CARMA2*sh* also requires the adapter molecule TRAF2 (20), although this evidence comes from experiments carried out in non-keratinocytic cell lines. In these cells, CARMA2*sh* expression protects cells from apoptosis induced by different stimuli, including ER stress (20).

The psoriasis-associated CARMA2*sh* mutants Glu138Ala and Glu142Gly also escape the negative regulation exerted by two novel CARMA2*sh* interactors identified by two-hybrid screening in yeast, namely the serine/threonine kinase Unc-51 Like Autophagy Activating Kinase 2 (ULK2) and the E3 ubiquitin ligase Ring Finger protein 7 (RNF7) (57–59). Both ULK2 and RNF7 are indeed able to repress CARMA2*sh*-induced NF-κB activation, although through different mechanisms. In particular, ULK2 phosphorylates CARMA2*sh* and promotes lysosomal degradation of BCL10, whereas RNF7 alters the ubiquitination state of MALT1 and NEMO (58, 59). Intriguingly, a protein similar to RNF7, named RNF181, has been identified as an interactor of CARMA1 and functions as an E3 ubiquitin ligase to inhibit antigen receptor signaling to NF-κB downstream of CARMA1 (60). Conversely, the ability of CARMA2*sh* to activate NF-κB is positively regulated by the DEP domain-containing protein DEPDC7, which may be required to specifically convey on the CBM complex signals coming from activated G protein-coupled receptors (61).

Recent evidence indicates that CARMA2*sh* and MALT1 play a role in the signal transduction pathway that connects pathogen-associated molecular patterns recognition to NF-κB activation (57, 58). Microorganisms, such as bacterial and fungi cells display in fact pathogen-associated molecular patterns (PAMPs), which are molecules associated with groups of pathogens, and activate NF-κB upon agonistic binding to Pattern Recognition Receptors, including members of the Toll-like receptors (TLR) family expressed on human keratinocytes. Indeed, depletion of each of the components of the CBM complex significantly impairs expression of NF-κB target genes in human epithelia keratinocytes exposed to heat-killed *Escherichia coli, Sthaphylococcus aureus* or *Candida valida* (58). Altogether, these findings strengthen the existence of a causal link between microbial infections and the onset of psoriasis and encourage more efforts in further clarifying how exposure to PAMPs could determine disruption of skin homeostasis, inflammation and hyperproliferation in susceptible keratinocytes.

## Animal models

Many interesting information can be inferred from the very recent generation of murine strains genetically modified in the *CARMA2* locus. Tanaka and colleagues generated CARMA2-deficient mice, which appear viable and healthy at birth and after, with normal epidermal and dermal tissue architecture (62). Interestingly, CARMA2^−/−^ mice are resistant to psoriasis provoked by intraperitoneal injection of recombinant IL-23 or treatment with imiquimod (IMQ) cream, an agonist of TLR7/TLR9. From these data, the authors suggest that CARMA2 is relevant for IL-23 receptor signaling in a population of IL-17- and IL-22-producing γδ T cells (62). The data, however, does not exclude the possibility that CARMA2 also controls the signaling cascade strarting from TLR7/TLR9.

In another study carried out by Mellet et al. (63), the psoriasic phenotype spontaneously develops in C57BL/6J mice harboring a CARMA2 mutation consisting in the deletion of a key glutamic acid residue at position 138 (CARMA2Δ138). Interestingly, while mice homozygous for E138 deletion show developmental abnormalities and die perinatally, heterozygous animals are viable, and a single copy of the gain-of-function mutation is sufficient to trigger psoriatic pathogenesis *in vivo*. Indeed, *CARD14*^Δ*E*138/+^ mice display all clinical and immunological features of chronic plaque-type psoriatic disease, with diffuse skin lesions characterized by epidermis thickening, persistence of hyperproliferating nucleated keratinocytes and immune cell infiltration in upper epidermal layers. In addition, affected skin from *CARD14*^Δ*E*138/+^ mice shows a transcriptome profile resembling the typical gene expression signature observed in human psoriatic plaques, including upregulation of hyperproliferative keratins, cytokines, chemokines and antimicrobial peptide-encoding genes. Consistently with previous findings, the pathologic skin phenotype displayed by this murine model is driven by the activation of IL23/IL17 axis, that promotes Th17 cell polarization via IL23, as confirmed by the fact that neutralization of IL23p19 with an antagonist antibody ameliorates disease symptoms, by reducing skin lesions and expression of inflammatory and anti-microbial genes (63). Similarly, the *CARD14*^*E*138*A*/+^ and *CARD14*^Δ*Q*136/+^ murine strains generated by Wang et al. also spontaneously develop psoriasis-like skin inflammation, which resulted from enhanced activation of the IL23/IL17 cytokine axis (64). Interestingly, these authors also show that CARMA2 associates with the ACT1-TRAF6 signaling complex, thereby mediating IL-17-induced NF-κB and MAPK signaling pathway activation, eventually responsible for expression of pro-inflammatory molecules.

## Conclusions

After establishing that *PSORS2* is due to NF-κB-activating mutations in *CARMA2*, current scientific advances are shedding some light on the molecular mechanisms that link these mutations to the development of human inflammatory skin diseases. Indeed, although for a long time CARMA2 was the most unknown of CARMA proteins, its clear involvement in the incipit and progression of inflammatory human skin disorders has acted as a strong propeller to clarify the biological and molecular processes in which this protein is involved. In many ways, CARMA2 acts just like the similar CARMA1 and CARMA3 proteins, with the CBM complex representing the molecular motor driving the signals transmission. However, some aspects of the signal tranduction pathways controlled by CARMA2 remain elusive. For example, it is not yet clear exactly what types of stimuli are channeled through CARMA2 to trigger NF-κB activation. Data generated in cell lines implicate CARMA2 in the signal transduction pathways starting from intracellular organelles, such as the endoplasmic reticulum, and from TLR receptors. In these contexts, CARMA2 activity seems to be controlled by TRAFs proteins and by molecules involved in autophagic processes, such as ULK2. Furthermore, ubiquitination mechanisms in which RNF and DEPDC7 proteins are involved seem to play a role in the capacity of CARMA2 to regulate the activity of NF-κB transcription factor.

The recent generation of murine strains harboring genetic modifications in the *CARMA2* locus is undoubtedly another important tool that will offer great opportunities to study in a complex biological system the role CARMA2 plays in the physiology and pathology of keratinocytes. Psoriasis is a typically human disease, and probably the lack of animal models that spontaneously develop the same disease has certainly represented a limit to our knowledge of this disease. Taking into account the obvious differences existing between human skin and murine skin, the fact that *CARMA2*-modified mouse strains develop inflammatory disorders with features largely overlapping to human psoriasis represents a real breaktrough. The phenotypic analyzes conducted on these mice indicate that the main alteration resides in the signal transduction along the IL23/IL17 cytokine axis, that would be ultimately responsible for the development of the psoriatic phenotype.

The coming years will undoubtedly be decisive in placing all the knowledge we have acquired so far on CARMA2 in a clearer and more coherent picture.

## Author contributions

TZ, IP, SV, RS, and PV reviewed the literature and wrote the manuscript.

### Conflict of interest statement

The authors declare that the research was conducted in the absence of any commercial or financial relationships that could be construed as a potential conflict of interest.

## References

[1] Blonska M Lin X. NF-κB signaling pathways regulated by CARMA family of scaffold proteins. Cell Res. (2011) 21:55–70. 10.1038/cr.2010.182 21187856 PMC3193407

[2] Scudiero I Vito P Stilo R. The three CARMA sisters: so different, so similar: a portrait of the three CARMA proteins and their involvement in human disorders. J Cell Physiol. (2014) 229:990–7. 10.1002/jcp.24543 24375035

[3] Bertin J Wang L Guo YJ Poyet J Srinivasula S Merriam S. CARD11 and CARD14 are novel caspase recruitment domain (CARD)/membrane-associated guanylate kinase (MAGUK) family members that interact with BCL10 and activate NF-kappa B. J Biol Chem. (2001) 276:11877–82. 10.1074/jbc.M010512200 11278692

[4] Wang L Guo Y Huang WJ Ke X Poyet JL Manji GA. Card10 is a novel caspase recruitment domain/membrane-associated guanylate kinase family member that interacts with BCL10 and activates NF-kappa B. J Biol Chem. (2001) 276:21405–9. 10.1074/jbc.M102488200 11259443

[5] Gaide O Martinon F Micheau O Bonnet D Thome M Tschopp J. Carma1, a CARD-containing binding partner of Bcl10, induces Bcl10 phosphorylation and NF-kappaB activation. FEBS Lett. (2001) 496:121–7. 10.1016/S0014-5793(01)02414-0 11356195

[6] McAllister-Lucas LM Inohara N Lucas PC Ruland J Benito A Li Q. Bimp1, a MAGUK family member linking protein kinase C activation to Bcl10-mediated NF-kappaB induction. J Biol Chem. (2001) 276:30589–97. 10.1074/jbc.M103824200 11387339

[7] Jiang C Lin X. Regulation of NF-κB by the CARD proteins. Immunol Rev. (2012) 246:141–53. 10.1111/j.1600-065X.2012.01110.x 22435552 PMC3339759

[8] Hara H Wada T Bakal C Kozieradzki I Suzuki S Suzuki N. The MAGUK family protein CARD11 is essential for lymphocyte activation. Immunity (2003) 18:763–75. 10.1016/S1074-7613(03)00148-1 12818158

[9] Egawa T Albrecht B Favier B Sunshine MJ Mirchandani K O'Brien W. Requirement for CARMA1 in antigen receptor-induced NF-kappa B activation and lymphocyte proliferation. Curr Biol. (2003) 13:1252–8. 10.1016/S0960-9822(03)00491-3 12867038

[10] Newton K Dixit V. Mice lacking the CARD of CARMA1 exhibit defective B lymphocyte development and impaired proliferation of their B and T lymphocytes. Curr Biol. (2003) 13:1247–51. 10.1016/S0960-9822(03)00458-5 12867037

[11] Greil J Rausch T Giese T Bandapalli OR Daniel V Bekeredjian-Ding I. Whole-exome sequencing links caspase recruitment domain 11 (CARD11) inactivation to severe combined immunodeficiency. J Allergy Clin Immunol. (2013) 131:1376–83. 10.1016/j.jaci.2013.02.012 23561803

[12] Stepensky P Keller B Buchta M Kienzler AK Elpeleg O Somech R. Deficiency of caspase recruitment domain family, member 11 (CARD11), causes profound combined immunodeficiency in human subjects. J Allergy Clin Immunol. (2013) 131:477–85. 10.1016/j.jaci.2012.11.050 23374270

[13] Dadi H Jones TA Merico D Sharfe N Ovadia A Schejter Y. Combined immunodeficiency and atopy caused by a dominant negative mutation in caspase activation and recruitment domain family member 11 (CARD11). J Allergy Clin Immunol. (2018) 141:1818–30.e2. 10.1016/j.jaci.2017.06.047 28826773

[14] Juilland M Thome M. Role of the CARMA1/BCL10/MALT1 complex in lymphoid malignancies. Curr Opin Hematol. (2016) 23:402–9. 10.1097/MOH.0000000000000257 27135977 PMC4900422

[15] Grabiner B Blonska M Lin P You Y Wang D Sun J. CARMA3 deficiency abrogates G protein-coupled receptor-induced NF-{kappa}B activation. Genes Dev. (2007) 21:984–96. 10.1101/gad.1502507 17438001 PMC1847715

[16] McAllister-Lucas L Ruland J Siu K Jin X Gu S Kim DS. CARMA3/Bcl10/MALT1-dependent NF-kappaB activation mediates angiotensin II-responsive inflammatory signaling in nonimmune cells. Proc Natl Acad Sci USA. (2007) 104:139–44. 10.1073/pnas.0601947103 17101977 PMC1766317

[17] Jiang T Grabiner B Zhu Y Jiang C Li H You Y. CARMA3 is crucial for EGFR-Induced activation of NF-κB and tumor progression. Cancer Res. (2011) 71:2183–92. 10.1158/0008-5472.CAN-10-3626 21406399 PMC3059846

[18] Li Z Qu L Dong Q Huang B Li H Tang Z. Overexpression of CARMA3 in non-small-cell lung cancer is linked for tumor progression. PLoS ONE (2012) 7:e36903. 10.1371/journal.pone.0036903 22615840 PMC3352848

[19] Xia ZX Li ZX Zhang M Sun LM Zhang QF Qiu XS CARMA3 regulates the invasion, migration, and apoptosis of non-small cell lung cancer cells by activating NF-κB and suppressing the P38 MAPK signaling pathway. Exp Mol Pathol. (2016) 100:353–60. 10.1016/j.yexmp.2015.10.004 26526492

[20] Scudiero I Zotti T Ferravante A Vessichelli M Vito P Stilo R. Alternative splicing of CARMA2/CARD14 transcripts generates protein variants with differential effect on NF-κB activation and endoplasmic reticulum stress-induced cell death. J Cell Physiol. (2011) 226:3121–31. 10.1002/jcp.22667 21302310 PMC3229840

[21] Harden J Lewis S Pierson K Suárez-Fariñas M Lentini T Ortenzio FS. CARD14 expression in dermal endothelial cells in psoriasis. PLoS ONE (2014) 9:e111255. 10.1371/journal.pone.0111255 25369198 PMC4219711

[22] Afonina IS Van Nuffel E Baudelet G Driege Y Kreike M Staal J. The paracaspase MALT1 mediates CARD14-induced signaling in keratinocytes. EMBO Rep. (2016) 17:914–27. 10.15252/embr.201642109 27113748 PMC5278603

[23] Stilo R Liguoro D Di Jeso B Formisano S Consiglio E Leonardi A. Physical and functional interaction of CARMA1 and CARMA3 with Ikappa kinase gamma-NFkappaB essential modulator. J Biol Chem. (2004) 279:34323–31. 10.1074/jbc.M402244200 15184390

[24] Xu X Zhang H. The immunogenetics of psoriasis and implications for drug repositioning. Int J Mol Sci. (2017) 18:E2650. 10.3390/ijms18122650 29292715 PMC5751252

[25] Michalek IM Loring B John SM. A systematic review of worldwide epidemiology of psoriasis. J Eur Acad Dermatol Venereol. (2017) 31:205–12. 10.1111/jdv.13854 27573025

[26] Chen H Poon A Yeung C Helms C Pons J Bowcock AM. A genetic risk score combining ten psoriasis risk loci improves disease prediction. PLoS ONE (2011) 6:e19454. 10.1371/journal.pone.0019454 21559375 PMC3084857

[27] Jordan CT Cao L Roberson ED Pierson KC Yang CF Joyce CE. PSORS2 is due to mutations in CARD14. Am J Hum Genet. (2012) 90:784–95. 10.1016/j.ajhg.2012.03.012 22521418 PMC3376640

[28] Jordan C Cao L Roberson E Duan S Helms C Nair R. Rare and common variants in CARD14, encoding an epidermal regulator of NF-kappaB, in psoriasis. Am J Hum Genet. (2012a) 90:796–808. 10.1016/j.ajhg.2012.03.013 22521419 PMC3376540

[29] Fuchs-Telem D Sarig O van Steensel M Isakov O Israeli S Nousbeck J. Familial pityriasis rubra pilaris is caused by mutations in CARD14. Am J Hum Genet. (2012) 91:163–70. 10.1016/j.ajhg.2012.05.010 22703878 PMC3397268

[30] Ammar M Jordan C Cao L Lim E Bouchlaka Souissi C Jrad A. CARD14 alterations in Tunisian patients with psoriasis and further characterization in European cohorts. Br J Dermatol. (2016) 174:330–7. 10.1111/bjd.14158 26358359 PMC5444086

[31] Körber A Mössner R Renner R Sticht H Wilsmann-Theis D Schulz P. Mutations in IL36RN in patients with generalized pustular psoriasis. J Invest Dermatol. (2013) 133:2634–7. 10.1038/jid.2013.214 23648549

[32] Eskin-Schwartz M Basel-Vanagaite L David M Lagovsky I Ben-Amitai D Smirin-Yosef P. Intra-familial variation in clinical phenotype of CARD14-related psoriasis. Acta Derm Venereol. (2016) 96:885–7. 10.2340/00015555-2405 26984337

[33] Takeichi T Sugiura K Nomura T Sakamoto T Ogawa Y Oiso N. Pityriasis rubra pilaris type V as an autoinflammatory disease by CARD14 mutations. JAMA Dermatol. (2017) 153:66–70. 10.1001/jamadermatol.2016.3601 27760266

[34] Craiglow B Boyden L Hu R Virtanen M Su J Rodriguez G. CARD14-associated papulosquamous eruption: a spectrum including features of psoriasis and pityriasis rubra pilaris. J Am Acad Dermatol. (2018) 79:487–94. 10.1016/j.jaad.2018.02.034 29477734 PMC6098739

[35] Takeichi T Kobayashi A Ogawa E Okuno Y Kataoka S Kono M. Autosomal dominant familial generalized pustular psoriasis caused by a CARD14 mutation. Br J Dermatol. (2017) 177:e133–5. 10.1111/bjd.15442 28295164

[36] Lwin S Hsu CK Liu L Huang HY Levell NJ McGrath JA. Beneficial effect of ustekinumab in familial pityriasis rubra pilaris with a new missense mutation in CARD14. Br J Dermatol. (2018) 178:969–72. 10.1111/bjd.15462 28301045 10.1111/bjd.15462

[37] Qin P Zhang Q Chen M Fu X Wang C Wang Z. Variant analysis of CARD14 in a Chinese Han population with psoriasis vulgaris and generalized pustular psoriasis. J Invest Dermatol. (2014) 134:2994–6. 10.1038/jid.2014.269 24999592 PMC4227539

[38] Eytan O Sarig O Sprecher E van Steensel M. Clinical response to ustekinumab in familial pityriasis rubra pilaris caused by a novel mutation in CARD14. Br J Dermatol. (2014) 171:420–2. 10.1111/bjd.12952 24641799

[39] Has C Schwieger-Briel A Schlipf N Hausser I Chmel N Rösler B. Target-sequence capture and high throughput sequencing identify a de novo CARD14 mutation in an infant with erythrodermic pityriasis rubra pilaris. Acta Derm Venereol. (2016) 96:989–90. 10.2340/00015555-2446 27140437

[40] Inoue N Dainichi T Fujisawa A Nakano H Sawamura D Kabashima K CARD14 Glu138 mutation in familial pityriasis rubra pilaris does not warrant differentiation from familial psoriasis. J Dermatol. (2016) 43:187–9. 10.1111/1346-8138.13008 26130407

[41] Sugiura K Muto M Akiyama M. CARD14 c.526G>C (p.Asp176His) is a significant risk factor for generalized pustular psoriasis with psoriasis vulgaris in the Japanese cohort. J Invest Dermatol. (2014) 134:1755–7. 10.1038/jid.2014.46 24476623

[42] Berki DM Liu L Choon SE David Burden A Griffiths CEM Navarini AA. Activating CARD14 mutations are associated with generalized pustular psoriasis but rarely account for familial recurrence in psoriasis vulgaris. J Invest Dermatol. (2015) 135:2964–70. 10.1038/jid.2015.288 26203641

[43] Mössner R Frambach Y Wilsmann-Theis D Löhr S Jacobi A Weyergraf A Palmoplantar pustular psoriasis is associated with missense variants in CARD14, but not with loss-of-function mutations in IL36RN in European patients. J Invest Dermatol. (2015) 135:2538–41. 10.1038/jid.2015.186 25989471

[44] Zhu K Shi G Liu H Zhu C Fan Y. Variants of CARD14 gene and psoriasis vulgaris in southern Chinese cohort. An Bras Dermatol. (2016) 91:45–8. 10.1590/abd1806-4841.20164016 26982778 PMC4782646

[45] Lau BW Lim DZ Capon F Barker JN Choon SE. Juvenile generalized pustular psoriasis is a chronic recalcitrant disease: an analysis of 27 patients seen in a tertiary hospital in Johor, Malaysia. Int J Dermatol. (2017) 56:392–9. 10.1111/ijd.13489 28194751

[46] Podlipnik S Castellanos-Moreira R Florez-Enrich H Arostegui JI Mascaró JM Jr. Acute generalized exanthematous pustulosis and polyarthritis associated with a novel CARD14 mutation. Australas J Dermatol. (2018) 59:e70–3. 10.1111/ajd.12669 28776328

[47] González-Lara L Coto-Segura P Penedo A Eiris N Díaz M Santos-Juanes J. SNP rs11652075 in the CARD14 gene as a risk factor for psoriasis (PSORS2) in a Spanish cohort. DNA Cell Biol. (2013) 32:601–4. 10.1089/dna.2013.2109 23905699

[48] Sugiura K Kitoh T Watanabe D Muto M Akiyama M. Childhood-onset PsA in Down syndrome with psoriasis susceptibility variant CARD14 rs11652075. Rheumatology (2015) 54:197–9. 10.1093/rheumatology/keu419 25342377

[49] Feng C Wang T Li SJ Fan YM Shi G Zhu KJ. CARD14 gene polymorphism c.C2458T (p.Arg820Trp) is associated with clinical features of psoriasis vulgaris in a Chinese cohort. J Dermatol. (2016) 43:294–7. 10.1111/1346-8138.13065 26249641

[50] Van Nuffel E Schmitt A Afonina IS Schulze-Osthoff K Beyaert R Hailfinger S CARD14-mediated activation of paracaspase MALT1 in keratinocytes: implications for psoriasis. J Invest Dermatol. (2017) 137:569–75. 10.1016/j.jid.2016.09.031 27939769

[51] Coto-Segura P González-Fernández D Batalla A Gómez J González-Lara L Queiro R. Common and rare CARD14 gene variants affect the antitumour necrosis factor response among patients with psoriasis. Br J Dermatol. (2016) 175:134–41. 10.1111/bjd.14461 26854129

[52] Wu K Reynolds N. CARD14 mutations may predict response to antitumour necrosis factor-α therapy in psoriasis: a potential further step towards personalized medicine. Br J Dermatol. (2016) 175:17–8. 10.1111/bjd.14795 27484270 PMC5961628

[53] Howes A O'Sullivan P Breyer F Ghose A Cao L Krappmann D. Psoriasis mutations disrupt CARD14 autoinhibition promoting BCL10-MALT1-dependent NF-κB activation. Biochem J. (2016) 473:1759–68. 10.1042/BCJ20160270 27071417 PMC5810350

[54] Sommer K Guo B Pomerantz JL Bandaranayake AD Moreno-García ME Ovechkina YL. Phosphorylation of the CARMA1 linker controls NF-kappaB activation. Immunity (2005) 23:561–74. 10.1016/j.immuni.2005.09.014 16356855

[55] Matsumoto R Wang D Blonska M Li H Kobayashi M Pappu B. Phosphorylation of CARMA1 plays a critical role in T Cell receptor-mediated NF-kappaB activation. Immunity (2005) 23:575–85. 10.1016/j.immuni.2005.10.007 16356856

[56] Stilo R Varricchio E Liguoro D Leonardi A Vito P. A20 is a negative regulator of BCL10- and CARMA3-mediated activation of NF-kappaB. J Cell Sci. (2008) 121:1165–71. 10.1242/jcs.021105 18349075

[57] Schmitt A Grondona P Maier T Brändle M Schönfeld C Jäger G. MALT1 protease activity controls the expression of inflammatory genes in keratinocytes upon zymosan stimulation. J Invest Dermatol. (2016) 136:788–97. 10.1016/j.jid.2015.12.027 26767426

[58] Scudiero I Mazzone P D'Andrea L Ferravante A Zotti T Telesio G. CARMA2sh and ULK2 control pathogen-associated molecular patterns recognition in human keratinocytes: psoriasis-linked CARMA2sh mutants escape ULK2 censorship. Cell Death Dis. (2017) 8:e2627. 10.1038/cddis.2017.51 28230860 PMC5386493

[59] Telesio G Scudiero I Pizzulo M Mazzone P Zotti T Voccola S. The E3 ubiquitin ligase RNF7 negatively regulates CARD14/CARMA2sh signaling. Int J Mol Sci. (2017) 18:E2581. 10.3390/ijms18122581 29194363 PMC5751184

[60] Pedersen SM Chan W Jattani RP Mackie DS Pomerantz JL. Negative regulation of CARD11 signaling and lymphoma cell survival by the E3 ubiquitin ligase RNF181. Mol Cell Biol. (2015) 36:794–808. 10.1128/MCB.00876-15 26711259 PMC4760215

[61] D'Andrea EL Ferravante A Scudiero I Zotti T Reale C Pizzulo M. The dishevelled, EGL-10 and pleckstrin (DEP) domain-containing protein DEPDC7 binds to CARMA2 and CARMA3 proteins, and regulates NF-κB activation. PLoS ONE (2014) 9:e116062. 10.1371/journal.pone.0116062 25541973 PMC4277425

[62] Tanaka M Kobiyama K Honda T Uchio-Yamada K Natsume-Kitatani Y Mizuguchi K. Essential role of CARD14 in murine experimental psoriasis. J Immunol. (2018) 200:71–81. 10.4049/jimmunol.1700995 29150564

[63] Mellett M Meier B Mohanan D Schairer R Cheng P Satoh TK. CARD14 gain-of-function mutation alone is sufficient to drive IL-23/IL-17-mediated psoriasiform skin inflammation *in vivo*. J Invest Dermatol. (2018) 138:2010–23. 10.1016/j.jid.2018.03.1525 29689250

[64] Wang M Zhang S Zheng G Huang J Songyang Z Zhao X. Gain-of-function mutation of card14 leads to spontaneous psoriasis-like skin inflammation through enhanced keratinocyte response to IL-17A. Immunity (2018) 49:66–79.e5. 10.1016/j.immuni.2018.05.012 29980436

